# Professor Germane and the Medical Experts

**Published:** 1891-11-21

**Authors:** Josiah Ritchie

**Affiliations:** Chairman. Royal Aquarium, Westminster


### Editor's Letter Box.

[Our correspondents are reminded that prolixity of statement is a great bar to publication, and that brevity of style and conciseness of statement greatly facilitate early insertion.]

### PROFESSOR GERMANE AND THE MEDICAL EXPERTS.

Sir,—The committee appointed by their medical confrères having to-day given their decision *re* Professor Germane, kindly favour me with space, in keeping with their permit to publish it, to explain the prior position. Yesterday week Professor Germane challenged (in the presence of over 1,000 medical gentlemen) his title to King Hypnotiser and the greatest mesmerist on earth. He invited all who would come upon the stage to test and use any means they liked to prove the genuineness of his performance. The stage becoming too crowded to thoroughly test Germane's power, those in the auditorium not being able to see, a committee of fifteen medical gentlemen, whose names are above suspicion, were appointed, who, with two exceptions, affirmed their belief in the genuineness of Professor Germane's hypnotic and mesmeric experiments. One gentleman, however, insisted that to more properly test Germane, he should attend a hospital, and there effect cures. To this Professor Germane readily assented, and a sub-committee of seven doctors was at once formed from the general committee of fifteen to carry out the arrangements. Publicity having so far been given, I am authorised to acquaint you with the result of the first and only deliberation of the sub-committee held here to-day.

Resolved : In view of the fact that an exhaustive committee of the British Medical Association is at present sitting to investigate the subject of hypnotism, this committee does not consider it desirable in the cause of medical science and the public interest to proceed further in the matter.

Recommended : That the British Medical Association accept Professor Germane's proferred services.

I may mention, in evidence of Professor Germane's courting the fullest inquiry, I had the addresses of the gentlemen forming the sub-committee] sent seriatim to the Chairman, and to each member of the committee I sent the Chairman's address. The members met in our board-room this morning, myself and Professor Germane being called in to receive their decision as above. I have not the slightest fault to find with it. I think they are correct in etiquette, and I hope the committee's recommendation that the British Medical Association will avail themselves of Professor Germane's services, will be accepted.

If Professor Germane's performance is not genuine, he must have in collusion with him the most laughable and valuable company of comedians ever seen on the face of the earth.—I am, sir, yours faithfully,

Josiah Ritchie, Chairman.

Royal Aquarium, Westminster,
November 3rd.

